# Endoscopic transgastric removal of a migrated intrahepatic fish bone via a cardia–lesser curvature tunnel

**DOI:** 10.1055/a-2800-4629

**Published:** 2026-04-20

**Authors:** Pingting Gao, Quan Zhou, Ping Zhou, Bing Li, Jiabin Cai, Mingyan Cai, Pinghong Zhou

**Affiliations:** 1 92323 Endoscopy Center and Endoscopy Research Institute, Zhongshan Hospital, Fudan University, Shanghai, China; 2 92323 Shanghai Collaborative Innovation Center of Endoscopy, Zhongshan Hospital, Fudan University, Shanghai, China; 3 92323 Key Laboratory of Carcinogenesis and Cancer Invasion, Ministry of Education, Liver Cancer Institute, Zhongshan Hospital, Fudan University, Shanghai, China

A 37-year-old man developed fever (to 40°C) and abdominal pain after inadvertent fish bone ingestion. A contrast-enhanced computed tomography scan showed a left-lobe liver abscess adjacent to a 3-cm linear hyperdense foreign body. Antibiotic therapy and percutaneous abscess drainage were performed.

**Fig. 1:**
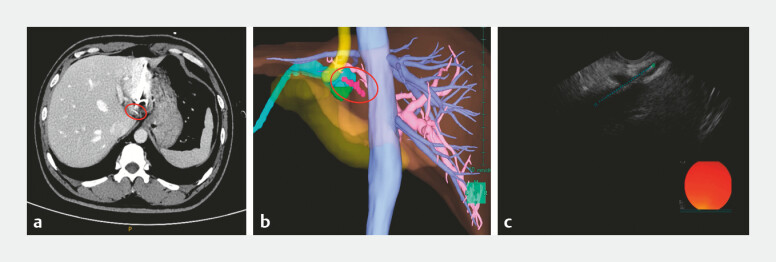
Preoperative imaging. **a** CECT showing the fish bone with adjacent abscess. **b** Annotated 3D reconstruction (the posterior to anterior view). **c** EUS confirming the foreign body tract and guiding the clip placement for endoscopic route. The fish bone is indicated by a red circle. CECT, contrast-enhanced computed tomography; EUS, endoscopic ultrasound.

After multidisciplinary discussion, a single-stage, entirely endoscopic natural-orifice transluminal endoscopic surgery (NOTES) was planned 1 . Computed tomography with 3D reconstruction localized the foreign body ( Fig. 1 ). Endoscopic ultrasound (EUS) confirmed a safe transgastric route and a metallic clip was placed to mark the optimal entry point.

**Fig. 2:**
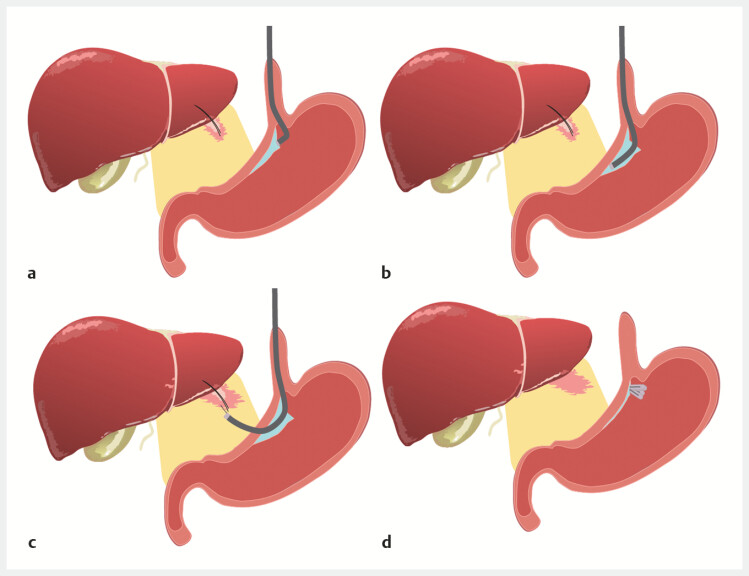
Schematic illustration of the procedure showing the creation of a submucosal tunnel, followed by the transgastric entry and retrieval of the intrahepatic fish bone.

**Fig. 3:**
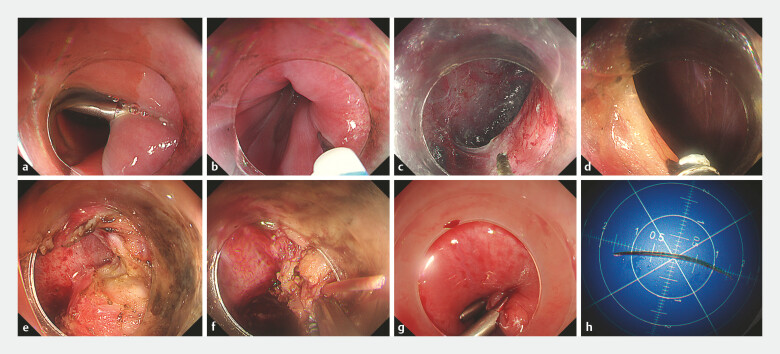
Endoscopic images showing key procedural steps. **a** Identification of the metallic clip placed under EUS guidance. **b** Submucosal injection of indigo carmine-stained saline. **c** Creation of a submucosal tunnel. **d** Entry into the peritoneal cavity. **e** Dissection of inflammatory adhesions. **f** Exposure and retrieval of the fish bone. **g** Closure of the tunnel entry with metallic clips. **h** The retrieved fish bone. EUS, endoscopic ultrasound.

**Fig. 4:**
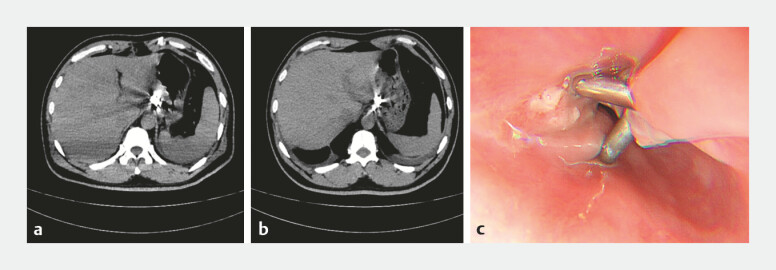
Postoperative imaging. **a** POD 2 CT demonstrating reduced inflammation with no complications. **b** POD 5 CT showing near-complete resolution of the abscess. **c** POD 5 endoscopy confirming intact clip closure and a well-healed mucosal entry. CT, computed tomography; POD, postoperative day.

The steps are as follows ( Fig. 2 and Fig. 3 ; Video 1 ): (1) Tunnel creation: A submucosal tunnel was established at the cardia 3 cm proximal to the EUS-placed clip. (2) Transgastric entry: At the distal end of the tunnel, a full-thickness myotomy was then made through the lesser curvature to enter the lesser sac. Upon entering the abdominal cavity via the lesser omentum, the foreign body on the visceral surface of the Spiegel lobe was promptly identified. (3) Intra-abdominal procedure: Meticulous dissection of inflammatory adhesions between omentum and the left hepatic lobe exposed the embedded fish bone. (4) Retrieval and closure: The foreign body was retrieved intact. Following irrigation, the myotomy site was coagulated and the tunnel entry securely. The whole procedure took 180 minutes. Recovery was uneventful. Follow-up endoscopy on postoperative day 5 confirmed secure gastric closure ( Fig. 4 ), and the patient was discharged the same day without complications.

Endoscopic transgastric removal of a migrated intrahepatic fish bone via a cardia–lesser curvature tunnel.Video 1

Sharp foreign bodies can perforate and migrate to the liver, causing persistent abscess 2 3 . This procedure marks the world’s first single-stage endoscopic removal of the intrahepatic foreign body, averting a major hepatectomy. This case also validates that the cardia–lesser curvature tunnel approach is safe and effective for targeting lesions on the visceral surface of the left hepatic lobe, expanding the anatomic applicability of NOTES techniques.

Endoscopy_UCTN_Code_TTT_1AO_2AL
